# A Bibliometric Analysis of the Global Research Trend in Child Maltreatment

**DOI:** 10.3390/ijerph15071456

**Published:** 2018-07-10

**Authors:** Bach Xuan Tran, Thang Van Pham, Giang Hai Ha, Anh Toan Ngo, Long Hoang Nguyen, Thuc Thi Minh Vu, Ha Ngoc Do, Vu Nguyen, Anh Tuan Le Nguyen, Tung Thanh Tran, Nu Thi Truong, Vuong Quan Hoang, Tung Manh Ho, Nhue Van Dam, Thu Trang Vuong, Hung Quang Nguyen, Huong Thi Le, Hoa Thi Do, Mackenzie Moir, Yoko Shimpuku, Meghnath Dhimal, Shalini Subash Arya, Tu Huu Nguyen, Suraj Bhattarai, Carl A. Latkin, Cyrus S.H. Ho, Roger C.M. Ho

**Affiliations:** 1 Institute for Preventive Medicine and Public Health, Hanoi Medical University, Hanoi 100000, Vietnam; ngotoananh85@gmail.com (A.T.N.); dennguyenle@gmail.com (A.T.L.N.); lethihuong@hmu.edu.vn (H.T.L.); dothihoa1954@yahoo.com (H.T.D.); 2 Bloomberg School of Public Health, Johns Hopkins University, Baltimore, MD 21205, USA; carl.latkin@jhu.edu; 3 Vietnam Young Physician Association, Hanoi 100000, Vietnam; huutu85@gmail.com; 4 Department of Pediatrics, Hanoi Medical University, Hanoi 100000, Vietnam; tsbsthang@yahoo.com; 5 Department of Emergency Medicine, Vietnam National Children’s Hospital, Hanoi 100000, Vietnam; 6 Institute for Global Health Innovations, Duy Tan University, Da Nang 550000, Vietnam; giang.ighi@gmail.com (G.H.H.); tung.ighi@gmail.com (T.T.T.); truongnu.ighi@gmail.com (N.T.T.); 7 National Hospital of Obstetrics and Gynecology, Hanoi 100000, Vietnam; 8 Department of Public Health Sciences, Karolinska Institutet, SE-171 77 Stockholm, Sweden; longnh.ph@gmail.com; 9 Center for Research and Training, Tam Anh Hospital, Hanoi 100000, Vietnam; vuminhthuc2010@yahoo.com.vn; 10 Youth Research Institute, Ho Chi Minh Communist Youth Union, Hanoi 100000, Vietnam; ngochayri@gmail.com; 11 Department of Surgery, Hanoi Medical University Hospital, Hanoi 100000, Vietnam; nguyenvu@hmu.edu.vn; 12 Center for Interdisciplinary Social Research, Thanh Tay University, Hanoi 100000, Vietnam; qvuong@ulb.ac.be; 13 Solvay Brussels School of Economics and Management, Centre Emile Bernheim, Université Libre de Bruxelles, Brussels B-1050, Belgium; 14 Institute of Philosophy, Vietnam Academy of Social Sciences, Hanoi 100000, Vietnam; tung.ho@wu.edu.vn; 15 Faculty of Graduate Studies, National Economics University, Hanoi 100000, Vietnam; sshpa.2017@gmail.com; 16 Sciences Po Paris, Campus de Dijon, 21000 Dijon, France; thutrang.vuong@sciencespo.fr; 17 Vietnam Czech Friendship Hospital, Hai Phong 180000, Vietnam; nguyenquanghungvcfh@gmail.com; 18 School of Public Health, University of Alberta, Edmonton, AB T6G 1C9, Canada; mackenzie.moir@gmail.com; 19 Graduate School of Medicine, Kyoto University, Kyoto 606-8501, Japan; shimpuku.yoko.5n@kyoto-u.ac.jp; 20 Nepal Health Research Council (NHRC), Kathmandu 44600, Nepal; meghdhimal@gmail.com; 21 Institute of Occupational Medicine, Social Medicine and Environmental Medicine, Goethe University, Frankfurt am Main 60323, Germany; 22 Institute of Chemical Technology, Mumbai 400 019, India; shalu.ghodke@gmail.com; 23 London School of Hygiene & Tropical Medicine, University of London, London WC1E 7HT, UK; surajbpkihs2012@gmail.com; 24 Department of Psychological Medicine, National University Hospital, Singapore 119228, Singapore; cyrushosh@gmail.com; 25 Department of Psychological Medicine, Yong Loo Lin School of Medicine, National University of Singapore, Singapore 119228, Singapore; hocmroger@yahoo.com.sg

**Keywords:** child maltreatment, scientometrics, child abuse and neglect, global mapping, bibliometric analysis

## Abstract

Child maltreatment remains a major health threat globally that requires the understanding of socioeconomic and cultural contexts to craft effective interventions. However, little is known about research agendas globally and the development of knowledge-producing networks in this field of study. This study aims to explore the bibliometric overview on child maltreatment publications to understand their growth from 1916 to 2018. Data from the Web of Science Core Collection were collected in May 2018. Only research articles and reviews written in the English language were included, with no restrictions by publication date. We analyzed publication years, number of papers, journals, authors, keywords and countries, and presented the countries collaboration and co-occurrence keywords analysis. From 1916 to 2018, 47,090 papers (53.0% in 2010–2018) were published in 9442 journals. Child Abuse & Neglect (2576 papers; 5.5%); Children and Youth Services Review (1130 papers; 2.4%) and Pediatrics (793 papers, 1.7%) published the most papers. The most common research areas were Psychology (16,049 papers, 34.1%), Family Studies (8225 papers, 17.5%), and Social Work (7367 papers, 15.6%). Among 192 countries with research publications, the most prolific countries were the United States (26,367 papers), England (4676 papers), Canada (3282 papers) and Australia (2664 papers). We identified 17 authors who had more than 60 scientific items. The most cited papers (with at least 600 citations) were published in 29 journals, headed by the Journal of the American Medical Association (JAMA) (7 papers) and the Lancet (5 papers). This overview of global research in child maltreatment indicated an increasing trend in this topic, with the world’s leading centers located in the Western countries led by the United States. We called for interdisciplinary research approaches to evaluating and intervening on child maltreatment, with a focus on low-middle income countries (LMICs) settings and specific contexts.

## 1. Introduction

The World Health Organization has defined child maltreatment as: “all forms of physical and/or emotional ill-treatment, sexual abuse, neglect or negligent treatment or commercial or other exploitation, resulting in actual or potential harm to the child’s health, survival, development or dignity in the context of a relationship of responsibility, trust or power” [1]. This global health issue has existed since ancient civilization [2]. However, finding a full or accurate definition of this social problem is a continuing challenge to researchers, institutions and even governments. Thus, the aims and scope of each paper in this topic are different because of the database [3]. Due to the various definitions, as well as the paper’s objectives, we decided to choose the World Health Organization (WHO) definition on child maltreatment.

Approximately 40 million children worldwide are the victims and/or witnesses of child domestic violence each year [1]. Child abuse can be considered a key factor that causes long-life physical and mental health problems for victims and family members. Moreover, an abused child could develop deep and persistent injuries during his/her entire life [4]. Some reports show that the effects of childhood physical abuse can lead to serious emotional and behavioral problems, aggression, violence or depression [5,6,7]. However, the availability of the data in this research remains a challenge [8]. Finkelhor et al. showed that one of the most cited figures was the probability of a child being sexually abused [9]. This proportion, nevertheless, is different in some scientific meta-analysis. For example, in North America, Gorey et al. found the probability of child abuse to be 12–17% for girls and 5–8% for boys in 1997 [10]. Likewise, Finkelhol et al. found the likelihood to be 1 among 9 girls and 1 among 53 boys in 2013 [9]. From an international perspective, Stoltenborgh et al. reported the probability of child abuse to be 18.0% for girls and 7.6% for boys in 2011 [11]; Pereda et al. reported the proportion of child abuse, particularly child sexual abuse, as 19.2% and 7.4% for girls and boys respectively [12]. Although the public awareness of child maltreatment problems has been rising in society in recent times [13], it varies among countries [14,15]. The evolution of child protection began in the late 19th century, yet at different times in each country and beginning at the non-government level. For example, the first child agency, the New York Society for the prevention of Cruelty to Children (NYSPCC), was founded in 1875, followed by the Liverpool and London Society for the Prevention of Cruelty to Children in the UK in 1883 and 1884; in the state of New South Wales in Australia in 1890, and Sweden was the first country introducing a ban on corporal punishment in 1979. By 2016, about 50 countries had prohibited all physical punishment to children. In the United States, 11 States passed Child Access Prevention law to prevent the youth to use or purchase firearms [16]. In India, due to the weakness of the Child Marriage Restraint Law in 1929, a new law banning child marriage was passed in December 2016. In the United States, the International Child Marriage Prevention and Assistance Act was introduced in July 2006, which called for federal efforts to fight child marriage [17]. Legislation about child abuse and protection received the global concern since 1924 with the first International Declaration on the Rights of the Child. Yet this was only the first guidance without legislative authority. Not until 1989, child maltreatment received the official international concern with the United Nations Convention on the Rights of the Child (UNCRC) launching. However, the implication of global legislation met some difficulties [18].

Child maltreatment interventions and policies require substantial empirical evidence with high impacts in different perspectives. One of the primary methods to objectively evaluate the impact of research articles is bibliometric analysis, referring to the method of using measurable information of publications to reveal how the knowledge in the research documents is utilized [19]. Bibliometric analysis of published scientific articles possibly determines the changes of concern for specific topics (child maltreatment in this case) through the growth rate of publications overtime. In addition, the evidence from this analysis can reflect the importance of research topics in national and global contexts via mapping networks of individuals and institutions involving different studies. However, currently, bibliometric studies on the child maltreatment topic are still constrained. Given the needs of evidence about the global trend of child maltreatment research, this study aims to explore the bibliometric overview on child maltreatment publications to understand their growth from 1916 to 2018. Information from this study will critically contribute to develop the research agenda in this field and allow advancing the theories and models for basic changes in global child maltreatment interventions.

## 2. Materials and Methods

### 2.1. Search Strategy

A cross-sectional study was designed to analyze the bibliography related to child abuse. The Web of Science was selected as the database. Although there were many definitions of child abuse [20], after reviewing some papers, we decided to choose the WHO’s conceptual definition of child maltreatment which also helped us to construct the search strategy [1]. To make sure that most of the results would be covered, we also added “child trafficking” and “child grooming” in our search query [21,22]. The search query was built by using the combination of words related to “child maltreatment”: child, maltreatment, abuse, violence, sexual, emotional, physical, trafficking, grooming (full strategy in Table S1) via the category “Topic” on the Web of Science. In our study, we have used the following terms interchangeably: child abuse and neglect, child maltreatment, child violence or child abuse. Only two types of research papers were found: research articles and research reviews. Other document types such as books, book chapters or data papers were excluded from this study. There was also a restriction in language as we chose the English items only. However, no publication date was applied to this report.

### 2.2. Data Extraction

**Figure 1:**
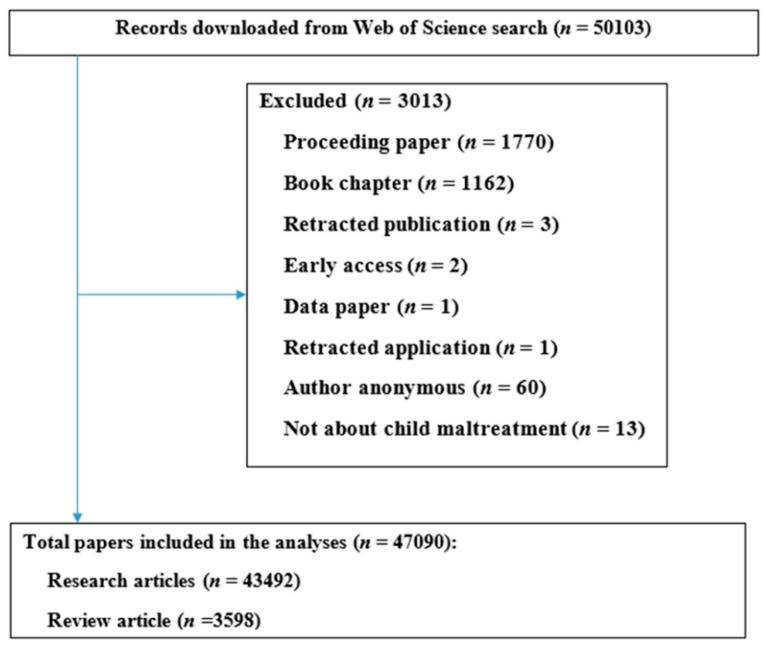
Selection of papers.

Data, which were downloaded online through the Web of Science, included authors’ names, the paper title, the journal name, keywords, institutional affiliations, frequency of citation, subject category, and abstract. Before downloading the data, articles were sorted by the frequency of citation. “Author keywords” and “keywords plus” were both used in this research. Additionally, we also downloaded the citation reports which were created automatically by the Web of Science. All of these data were stored in Microsoft Excel. After that, we filter all the downloaded data by excluding the papers which are: (1) not original articles and reviews; and (2) not about child abuse. A total of 3013 papers were excluded after screening titles and abstracts (Figure 1).

### 2.3. Data Analysis

**Figure 2:**
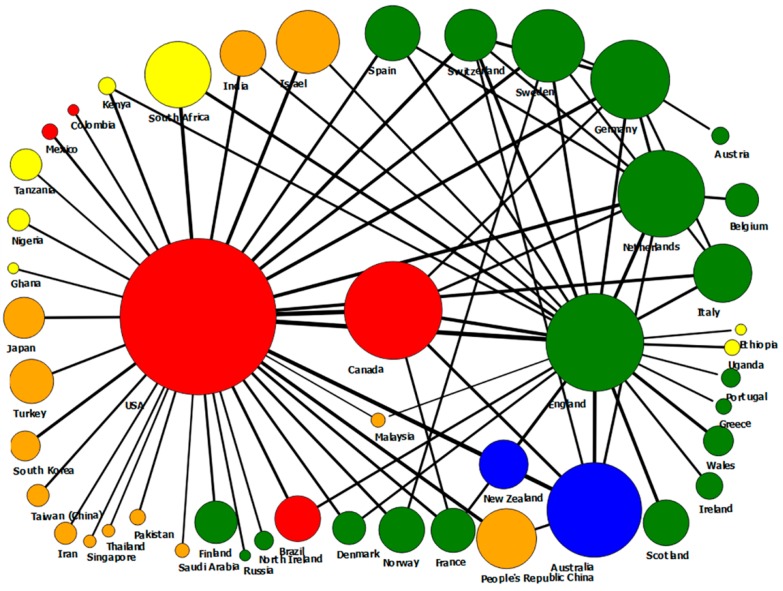
A global network of the most prolific countries (more than 50 papers). Note: America = Red; Europe = Green; Blue = Australia; Asia = Yellow, Africa & Middle East = Yellow.

Data were analyzed including: the basic characteristics (number of authors, publication years, main category), keywords (most common keywords and co-occurrence keywords), most prolific authors (more than 60 papers, countries with significant among of publications (more than 75 papers), chief journals, and highly impactful papers (times cited—more than 600 times). A network graph illustrates the connection among countries by applying the specific threshold of 50 papers for each collaboration (Figure 2). To find the most frequent keywords appearing in the keyword plus list, we used Wordle (http://www.wordle.net/) as it helped us to create a word cloud online and download the results directly. The larger the words within the cloud, the more often the keywords were repeated. Besides, the author keyword co-occurrence network was also created, which reflects the frequency of the keywords and the proportion of co-words. We used VOSviewer (version 1.6.8, Center for Science and Technology, Leiden University, the Netherlands), free software for creating a co-occurrence network. For countries network, we used Social Networks Visualiser (SocNetV (version 2.4, the Free Software Foundation, Massachusetts, the United States)), a user-friendly and free software tool for Social Network Analysis and Visualization. Data on population were obtained from the World Bank website with the total population of 2016 (https://data.worldbank.org/indicator/SP.POP.TOTL). However, the 2016 population of Wales, Scotland and Northern Ireland was collected from the Office for National Statistics Great Britain (https://www.ons.gov.uk/peoplepopulationandcommunity/populationandmigration/populationestimates).

## 3. Results

### 3.1. Number of Published Items and Publication Trend

A total of 47,090 research papers (43,492 articles and 3598 reviews) were included in the analysis, while 3013 documents that did not match eligible criteria were excluded (Figure 1).

Table 1 shows the characteristics of the papers. 47,090 papers were published by 9442 journals. 6478 (68.6%) journals published one paper, 808 journals published two, 393 (4.2%) journals published three and 1736 (18.7%) journals published four or more. Two journals that published the most papers were Child Abuse and Neglect (*n* = 2576; 5.5%) and Children and Youth Services Review (*n* = 1130; 2.4%), followed by Pediatrics (*n* = 793; 1.7%), Journal of Interpersonal Violence (*n* = 661; 1.4%) and Journals of Family Violence (*n* = 518; 1.1%). Most of the papers (82.7%) were sorted into one (*n* = 23,869; 50.7%) or two (*n* = 15,077; 32%) subject categories of the journals. Psychology (*n* = 16,049; 34.1%) and family studies (*n* = 8225; 17.5%) were two main subject categories.

Table 2 displayed in detail the most productive subject categories, with more than 500 papers, as well as the journals in which they were published. Child Abuse & Neglect was the leading journal with regards to the number of papers in these subjects: Psychology, Family Studies, and Social work. Among all topics, Child maltreatment was the one which attracted the huge concern of the community that could be clearly seen from the diversity of the subject categories, such as psychology, family studies, pediatric or government law, legal medicine or criminology penology.

### 3.2. Author and Countries

As can be seen in Table 1, nearly 40% (*n* = 18,526) of the papers were the work of 2 or 3 authors; the number of papers with 4 or 6 authors was 14,789 (31.4%), and only 11.5% (*n* = 665) items were written by seven or more authors. About 80.2% (*n* = 37,769) of total papers belonged to the first authors from North America and Europe. The first authors of the United States contributed 26,377 (56%) papers.

The results in Table 3 indicate the most productive authors with more than 60 papers during the time of the study. The American authors are amongst the most prolific, with ten authors who contributed much to this research area. Dante Cicchetti from the University of Minnesota, United States led the number of published papers (159 publications), as well as the number of papers in collaboration. However, Bert Brunekreef (Wageningen University & Research, Netherlands) was the author who received the most citations per paper.

Table 4 illustrates the productivity ranking of 192 countries involved in the sample of papers. In this list, the top five countries were from North America, Europe, and Australia. It appears that the United States was the knowledge hub of the world in this field. They ranked top in all indices: total papers (26,371 papers, 56%), total collaborations (9487 organizations), total citations (nearly eight hundred thousand), and countries in collaboration (169 countries). They were also the main collaborators of 37 countries (74%) in this list. England was in second place with 4676 papers (9.9%) and was the main collaborator of the countries in the United Kingdom and five other countries in Europe and Africa. The People’s Republic China was the only Asian country on the list of 10 leading countries in the number of total papers. However, they produced only 0.55 articles per one million inhabitants, which made them rank last in the list of papers per million inhabitants. New Zealand was ranked first in this list with 129.55 papers per million inhabitants.

Figure 2 displays the global network between 18 countries (with at least 50 papers in co-authorship), in which the connectivity of those countries and the position of each country in the network can be easily discovered. The size of nodes shows the proportional contribution to the number of papers and the thickness of lines indicates the percentage of the number of collaboration.

**Figure 3:**
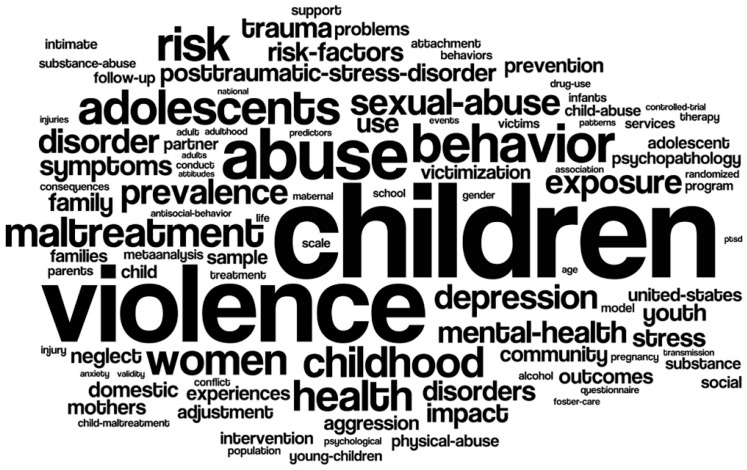
The most frequent keywords.

Figure 3 reveals the most common keywords which appeared more than 700 times in the literature (automatically counted by the Wordle); “children” was the most common word, appearing in 10,975 papers (23%), followed by “violence”, “abuse”, “behavior” and “adolescents” which occurred in 8931 papers (19%), 5890 papers (13%), 4493 papers (10%), and 4013 papers (9%) respectively.

**Figure 4:**
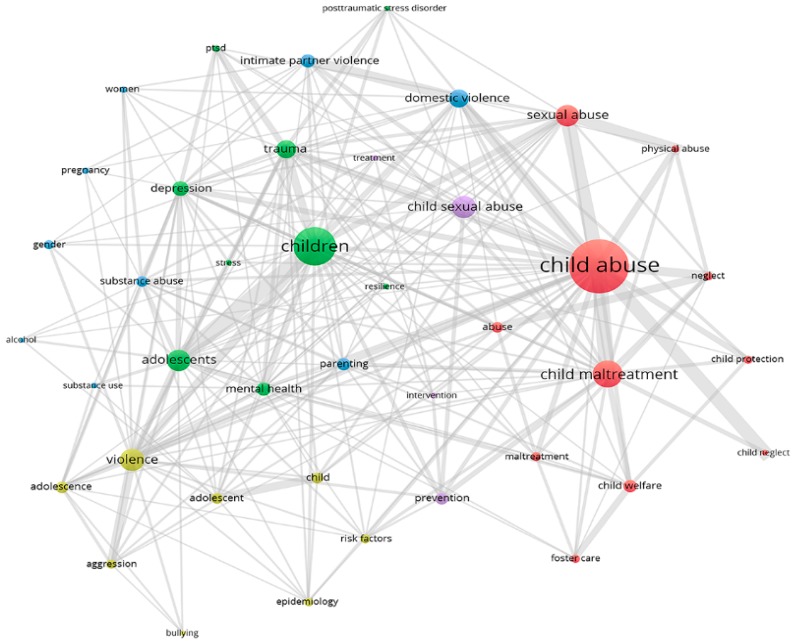
Co-occurrence of author’s keyword. Note: the colors of the nodes were automatically assigned by the software; the nodes’ size was scaled to the keywords’ occurrences; the thickness of the lines was automatically drawn by the software based on the strength of two keywords.

Figure 4 points out the keyword co-occurrence which is a measure of the probability that a particular word appears on the Web of Science search results. As can be seen in the figure, “child abuse” and “children” were two common words coming out the most amongst authors’ keywords. The thickness of the lines indicates the strength of one given keyword relative to the others, such as, the association of “child abuse” and “children” with “mental health”, “parenting”, “violence” or “depression”; the association of “prevention” with “child maltreatment”, “child sexual abuse”, “child abuse” or “intervention”. Looking at Figure 4, we identified the main groups, including: (1) research subjects (children, adolescents, child and women); (2) types of maltreatment (child abuse, sexual abuse, physical abuse, neglect, maltreatment, violence, bullying); (3) health outcomes (post-traumatic stress disorder (PTSD), stress, depression, pregnancy, mental health, and trauma); (4) risk factors (substance abuse, substance use, and alcohol); and (5) child protection (intervention, prevention, and treatment).

### 3.3. Most Cited Papers

The list of papers with the highest number of citations can be found in Table 5. In total, all papers included in this research together received about 1.1 million citations, of which 2022 papers have more than 100 citations. Those most highly cited papers (48 papers in the list) were published in 29 journals, headed by JAMA Journal of the American Medical Association with seven papers, followed by the Lancet, containing five papers. This list also shows the comprehensive review of research on the following topics: the effect of neighborhood or family and community on the children (paper number 7 and paper number 47 respectively in Table 5); the survey and research about child abuse (paper number 42, number 46 and number 48 in Table 5); the effect of child abuse (paper number 6, number 10, number 14, number 15, number 18, number 28, number 29, number 34 and number 39 in Table 5); the investigation in bullying and the role of schoolboys and schoolgirls within the group process in a Finnish school (paper number 35 in Table 5); and the negative outcomes of bullying among the US youth, in paper number 5, as well as suggestions regarding preventing interventions.

## 4. Discussion

In this scientometrics research, we focused on analyzing the international science and scientific reports on child maltreatment during the period 1916–2018 (May 2018). We have recognized the most dynamic authors and countries, most frequent subject areas, as well as common keywords, most productive journals, and citation reports in child abuse and maltreatment, based on the research publications on the Web of Science Core Collection. There have been an increasing number of published items in the last decades, with more than half of the reports published since 2010. To the best of our understanding, this can be considered the first inclusive global mapping and analysis of scientific research papers in child maltreatment. In agreement with some research in different areas, our research has also confirmed that the United States was the science hub of knowledge-sharing and the global leader in research publications in this discipline [23,24], followed by the countries in Western Europe (such as England, the Netherlands and Germany), Canada and Australia. The large amount of research on child abuse from these countries reflects the huge concern that the Western world has dedicated to find the means and prevalence of child abuse, as well as the effects of child maltreatment on youth society. Additionally, it reflects that awareness of child abuse is on the rise. However, some reports showed that the trend of child abuse was “not a true increase in prevalence but due to the changes in legislation” [25,26]. In another context, some researchers found that there were no changes in the rate of child physical abuse [27,28], even though there was a rise in the prevalence of child emotional abuse [28].

The most powerful collaborations worldwide occurred among authors and organizations from the United States, England and Canada. We predicted the limited contribution of the authors in low-and-middle-income countries, especially in Asia, partly due to the concept of societies that considered child sexual abuse as “sensitive and taboo” and partly due to the adult’s conceptualization of the need of child punishment for raising children [29,30]. For example, in India, child sexual abuse was historically a hidden problem and usually ignored by the public [31]. This public health problem was brought to international attention only since 1988 [32]. In 2012, a new Bill on the ‘Protection of Children from Sexual Offences’ became an Act [31]. In developed countries, governments took a leadership role early on, such as the U.S Congress passing the “Child Abuse Prevention and Treatment Act” (CAPTA) in 1974, and in England the “Children Act 1989”provided the legislative framework for child protection and was later strengthened by the Children Act 2004. Another valuable example here is in European countries. Although each European country has different political systems, the countries share a common mission: child protection. At the European Union (EU) level, the European children’s rights law was submitted by the Council of Europe (CoE) and the EU. Recently, children’s rights have been considered as an important issue which needs more cooperation [33]. One explanation for such a discrepancy among geo-social regions could be the variation in mindsets among the people. For example, in India, there are a number of deep-rooted socio-culture elements; gender discrimination caused by male child preference being the most worrisome one [34]. Another noteworthy example that can be considered is corporal punishment, which differs among countries’ cultures and child–rearing practices. According to a research paper with a sample of nine countries, the use of corporal punishment on children was lowest by Swedish parents and highest by Kenyan parents [35]. A similar pattern has been observed with Southeast Asian countries; there is a cultural norm in Vietnamese parents: “spare the rod, spoil the child” and they usually use harsh discipline to raise a well-mannered child [36].

Journals that published the most papers related to child maltreatment were Child Abuse & Neglect and Children and Youth Services Review. In fact, the most commented-upon topic of child maltreatment was child neglect, and it can be explained that this is the most common form of child maltreatment [37]. Our analysis revealed that nearly 51.6% of all scientific papers were published in the research discipline of psychology and family studies. A large number of papers in psychology and family studies could be attributed to the view that family education is important to prevent child abuse [38] and/or the child’s normal emotional development is interrupted due to abuse victimization [39]. Therefore, in this topic, the development of psychology can bring a positive impact on increasing research and intervention [40]. Our study concludes that child maltreatment has triggered a deep concern among research authors, who performed research in various disciplines, from Government Law [41,42], Social Sciences [43], Criminology Penology [44], Neurosciences [45], to General Internal Medicine [46,47,48]. The authors’ keywords co-occurrence analysis has explained the level of concern about this topic. For example, “child abuse” was the most regular keyword that has strong connection to common types of child maltreatment: sexual abuse, physical abuse, and neglect. Besides, “domestic violence” (among parents or other family members) with the child witnessing was also considered child abuse, as it has direct the effects on the child’s overall development [49]. The number of papers in child protection, intervention, and child abuse prevention contributed a small number compared with that of child abuse or child maltreatment. Likewise, the epidemiology of child maltreatment was more readily found in high-income countries than in low-and-middle-income countries (LMICs). Our result has been in line with the report prepared by the United Nations Children Fund (UNICEF), in which stress has been found to be empirically associated with child abuse, and alcohol and substance abuse have contributed to the higher risk [50].

The list of most cited research articles showed us the research interest in topics on child maltreatment. The effects of child maltreatment [51,52,53] and research about child abuse and violence [54,55] attracted audiences to the authors the most. However, there is a huge knowledge gap in these research fields [56,57], such as the effectiveness of child abuse and neglect interventions, especially in low-and-middle-income countries (LMICs). Despite the fact that the developed countries have over 30 years of trend in evaluating the effectiveness of intervention programs, the knowledge may not be relevant or applicable in LMICs. Part of the reason can be the priority that the LMICs’ governments set for spending to adapt basic needs (food supply, electricity or water) and reduce poverty and crime or the lack of investment in research and development (R&D). Thus, LMICs may have adapted the intervention model of high-income countries without adequate or necessary and sufficient conditions for successful interventions. From our point of view, LMICs have advantages when they can inherit some useful models from high-income countries, provided they train local experts to the extent that professionals carefully appreciate the cultures and efficiently connect to the knowledge hubs. 

We must discuss some weakness and limitations of our study. First, the Web of Science database was the only one we used to generate the scientific papers, including the characteristics of the data (such as the number of records, publication year or authors) and for examining the network of keywords and international collaboration. However, articles that belong to open access data not provided by the Web of Science may affect the search results [58]. Secondly, the analysis of keywords co-occurrence depends on the chosen keywords; and this can be a source of bias toward a cohort of the Web of Science-indexed papers.

Thirdly, due to the vastly different definitions of child abuse and neglect in different organizations and countries, it is sometimes difficult to choose the one which can satisfactorily cover all forms of child maltreatment. For example, the types of child abuse and neglect are recognized differently by different States in the United States. Although most States recognize physical abuse, neglect, sexual abuse, and emotional abuse, some states also consider parental substance abuse and/or child abandonment as forms of child abuse [59]. Besides, with the rapid development of technology, a new form of child abuse has recently been mentioned in a report of the National Society for the Prevention of Cruelty to Children (NSPCC): online abuse [60]. In addition, UNICEF and INSPIRE program (WHO) reports show that there are some forms of violence which are harmful to children that are outside of the child abuse definition, such as child bullying, children in armed conflict, child soldiers, or the homicide of street children [8,16]. More than that, in the form of foster care, children may have suffered some forms of maltreatment and some characteristics are identified to predict the risks [61]. What we followed was the WHO report that has defined four types of child maltreatment: child sexual abuse, child physical abuse, child emotional abuse and child psychological abuse [1]. Besides, we performed the search query by applying the notions of child violence, child trafficking and child grooming [21,22]. As we did not use previously mentioned words, our search results may be not fully adequate. Therefore, there is a need for more cooperation among researchers, organizations, and governments to reach a more ‘universal’ and ‘generally accepted’ definition of this serious social problem.

As we included only research articles and reviews, a significant number of important reports, such as progress reports, annual reports of governments, books or book chapters, and guidance from governments representing an excellent contribution to the to the topic of child abuse and violence should also be considered for a more complete evaluation [59,60,62]. Human language is extremely diverse [63], but we included only English articles and reviews in our study; thus, non-English research papers in Europe, Asia or Africa are not counted. That made the number of Western countries’ publications, especially in English speaking ones, more than that of the Asia or Africa regions. Finally, although we have tried to control the quality of the research results by reading titles and abstracts only, sometimes there are papers with less relevant contents still appearing in the review workload. Fortunately, they only count a small fraction and should not seriously affect the results. Having appreciated the attention from not only the scientific circles, but also the public, governments and educators, we conclude that this topic in the coming years will be high on various agendas, with a strong emphasis being given on the highly practical subfields of prevention and intervention of child maltreatment.

## 5. Conclusions

The scientometrics analysis has provided highly relevant data for evaluating the growth of scientific research on child maltreatment. The number of research papers published has been increasing with the most influential knowledge and ideas coming from Western countries; notably, the United States has led the trend. This research made a contribution to the extant literature regarding this important social problem, and recognized the main research areas, publications and leading scientific authors and countries. Development in research methods and extension in the research areas will allow for the evaluating of relevant research methods, and would likely contribute to child abuse prevention, as well as future agendas for the cause of public health improvements.

## Figures and Tables

**Table 1:** Characteristics of selected articles.

Characteristic	Category	Number	Percent
Total number of papers		47,090	100.0
Year of publication			
	1916–1979	500	1.1
	1980–1989	1113	2.4
	1990–1999	7066	15.0
	2000–2009	13,464	28.6
	2010–2018	24,947	53.0
Number of authors			
	1	8398	17.8
	2–3	18,520	39.3
	4–6	14,787	31.4
	7–10	4265	9.1
	>10	1120	2.4
Number of subject categories			
	1	23,869	50.7
	2	15,077	32.0
	3	7484	15.9
	4	563	1.2
	>5	97	0.2
Main subject categories			
	Psychology	16,049	34.1
	Family studies	8225	17.5
	Social work	7367	15.6
	Psychiatry	6365	13.5
	Pediatrics	4938	10.5
	Others	4146	8.8
Country of the first author (top 10)			
	United States	26,371	56.0
	England	4675	9.9
	Canada	3282	7.0
	Australia	2664	5.7
	Netherlands	1244	2.6
	Germany	1188	2.5
	Sweden	1002	2.1
	South Africa	911	1.9
	Israel	860	1.8
	The People’s Republic of China	755	1.6

**Table 2:** Journal subject categories and the journals which included those subject categories.

Journal Subject Category	Total Papers	Journal Name	Total Papers	Journal Name	Total Papers
Psychology	16,049	Child Abuse & Neglect	2576	Journal of The American Academy of Child And Adolescent Psychiatry	492
		Journal of Interpersonal Violence	661	Journal of Child Sexual Abuse	377
		Journal of Family Violence	518	Development and Psychopathology	327
Family studies	8225	Child Abuse & Neglect	2576	Journal of Family Violence	518
		Children and Youth Services Review	1130	Journal of Child Sexual Abuse	377
		Journal of Interpersonal Violence	661	Child Maltreatment	362
Social work	7367	Child Abuse & Neglect	2576	Child Welfare	355
		Children and Youth Services Review	1130	American Journal of Orthopsychiatry	304
		Child Maltreatment	362	British Journal of Social Work	255
Psychiatry	6365	Journal of The American Academy of Child and Adolescent Psychiatry	492	Journal of Traumatic Stress	190
		American Journal of Orthopsychiatry	304	Journal of Child Psychology and Psychiatry	164
		Journal of Child and Family Studies	238	Child and Adolescent Psychiatric Clinics of North America	151
Pediatric	4938	Pediatrics	793	Archives of Disease in Childhood	211
		Journal of The American Academy of Child and Adolescent Psychiatry	492	Pediatric Emergency Care	198
		Journal of Adolescent Health	246	Archives of Pediatrics Adolescent Medicine	171
Public environmental occupational health	4937	Journal of Adolescent Health	246	American Journal of Public Health	137
		BMC Public Health	242	Journal of Community Psychology	136
		Social Science Medicine	200	Accident Analysis and Prevention	121
Criminology penology	2642	Journal of Interpersonal Violence	661	International Journal of Offender Therapy and Comparative Criminology	117
		Aggression and Violent Behavior	242	Trauma Violence Abuse	113
		Violence and Victims	126	Criminal Justice and Behavior	112
General & internal medicine	2149	American Journal of Preventive Medicine	121	JAMA Journal of The American Medical Association	78
		Lancet	116	BMJ Open	67
		British Medical Journal	89	Journal of Trauma: Injury Infection and Critical Care	60
Neurosciences neurology	1822	Journal of Affective Disorders	113	Psychoneuroendocrinology	47
		Journal of Nervous and Mental Disease	94	Neurotoxic Ology and Teratology	34
		Biological Psychiatry	82	Childs Nervous System	33
Substance abuse	1589	Drug and Alcohol Dependence	134	Addiction	102
		Addictive Behaviors	117	Journal of Child & Adolescent Substance Abuse	99
		Journal of Substance Abuse Treatment	103	Substance Use & Misuse	94
Government law	1475	Behavioral Sciences & The Law	107	Law and Human Behavior	76
		Psychology Crime Law	84	Psychology Public Policy and Law	76
		Juvenile and Family Court Journal	77	Psychiatry Psychology and Law	71
Social sciences other topics	1217	Accident Analysis and Prevention	121	Childhood a Global Journal of Child Research	62
		Archives of Sexual Behavior	82	Child Indicators Research	48
		Future of Children	63	Evaluation and Program Planning	44
Education & educational research	1215	Journal of School Health	90	Research in Developmental Disabilities	34
		Journal of Emotional and Behavioral Disorders	46	Journal of Intellectual Disability Research	29
		Journal of Drug Education	41	Health Education Research	24
Health care sciences services	1004	Journal of School Health	90	Future of Children	63
		Psychiatric Services	76	Community Mental Health Journal	48
		Psychology Public Policy and Law	76	AIDS Care Psychological and Socio-Medical Aspects of AIDS/HIV	44
Environmental sciences ecology	974	International Journal of Environmental Research and Public Health	105	Environmental Research	85
		Science of the Total Environment	96	Atmospheric Environment	80
		Environmental Health Perspectives	91	Environmental Health	36
Nursing	863	Journal of Advanced Nursing	53	Journal of Pediatric Health Care	36
		Public Health Nursing	52	Nursing Clinics of North America	35
		Journal of Clinical Nursing	45	Revista Latino Americana de Enfermagem	29
Surgery	704	Journal of Trauma: Injury Infection and Critical Care	60	Journal of Pediatric Surgery	37
		Injury-International Journal of The Care of The Injured	58	Childs Nervous System	33
		Burns	40	Pediatric Surgery International	28
Sociology	648	Journal of Marriage and Family	56	Deviant Behavior	29
		Youth Society	34	Journal of Health And Social Behavior	18
		Journal of Marriage and The Family	31	Social Forces	18
Biomedical social sciences	594	Social Science Medicine	200	AIDS and Behavior	29
		AIDS Care Psychological and Socio-Medical Aspects of AIDS/HIV	44	Culture Health Sexuality	21
		Qualitative Health Research	34	Journal of Biosocial Science	20
Legal medicine	561	Forensic Science International	120	Journal of Forensic and Legal Medicine	71
		Journal of Forensic Sciences	112	Medicine Science and The Law	53
		American Journal of Forensic Medicine and Pathology	84	International Journal of Legal Medicine	44
Women studies	545	Violence against Women	156	Women Therapy	33
		Affilia: Journal of Women and Social Work	47	Journal of Women’s Health	31
		Sex Roles	34	Women’s Studies International Forum	29
Rehabilitation	538	Arts in Psychotherapy	42	Journal of Applied Research in Intellectual Disabilities	24
		Research in Developmental Disabilities	34	Sexuality and Disability	24
		Journal of Intellectual Disability Research	29	Brain Injury	20
Emergency medicine	524	Pediatric Emergency Care	198	Emergency Medicine Journal	31
		Injury-International Journal of The Care of The Injured	58	Annals of Emergency Medicine	30
		Journal of Emergency Medicine	32	Academic Emergency Medicine	25
Infectious disease	505	PLOS Neglected Tropical Diseases	123	International Journal of STD & AIDS	24
		BMC Infectious Diseases	28	AIDS	22
		International Journal of Hygiene and Environmental Health	27	Journal of Acquired Immune Deficiency Syndromes	22
Obstetrics & Gynecology	504	BMC Pregnancy and Childbirth	34	American Journal of Obstetrics and Gynecology	22
		Journal of Women’s Health	31	BMC Women’s Health	22
		Journal of Pediatric and Adolescent Gynecology	29	International Journal of Gynecology & Obstetrics	22

**Table 3:** Most prolific authors.

No	Author	Affiliation and Country	Total Papers	Total Citations	Citations per Paper	Papers in Collaboration	Total Signatures	Collaboration Index (Signatures per Paper)
1	Dante Cicchetti	University of Minnesota, United States	159	11,237	70.7	151	566	3.6
2	David Finkelhor	University of New Hampshire, United States	116	11,602	100.0	107	377	3.3
3	Howard Dubowitz	University of Maryland School of Medicine, United States	96	3166	33.0	87	531	5.5
4	Michael E. Lamb	University of Cambridge, England	96	3988	41.5	91	366	3.8
5	David M. Fergusson	University of Otago, New Zealand	87	8781	100.9	87	260	3.0
6	Joel S Milner	Northern Illinois University, United States	83	2061	24.8	76	335	4.0
7	John L. Horwood	Univ Otago Christchurch, New Zealand	77	6919	89.9	77	240	3.1
8	Cathy Spatz Widom	CUNY John Jay Coll Criminal Justice, United States	73	7354	100.7	65	185	2.5
9	Gail S. Goodman	University of California Davis, United States	71	2627	37.0	70	384	5.4
10	Martine B. Powell	Deakin University, Australia	70	654	9.3	69	212	3.0
11	Fred A. Rogosch	University of Rochester, United States	68	3627	53.3	68	246	3.6
12	Jürg Utzinger	Swiss Tropical & Public Health Institute, University of Basel, Switzerland	68	2231	32.8	68	694	10.2
13	Bert Brunekreef	Wageningen University & Research, Netherlands	65	6761	104.0	65	1528	23.5
14	Kenneth A. Dodge	Duke University, United States	65	4891	75.2	61	451	6.9
15	Harriet L. MacMillan	McMaster University, Canada	65	2732	42.0	63	345	5.3
16	Desmond K. Runyan	University of North Carolina, United States	64	2548	39.8	64	401	6.3
17	Penelope K. Trickett	University of Southern California, United States	61	2827	46.3	61	237	3.9

**Table 4:** Most prolific countries and the collaborations.

Country	Total Papers	Papers per Million Inhabitants	Total Collaborations	Total Citations	Citations per Paper	Papers in Collaboration (Distinct Country)	Distinct Countries of Collaboration	Main Collaborator (and Number of Collaborations)
USA	26,377	81.63	9487	784,603	29.75	3784	169	Canada (683)
England	4675	84.61	3990	124,144	26.55	2051	143	USA (682)
Canada	3282	90.50	2713	81,262	24.76	1182	130	USA (683)
Australia	2664	110.03	2506	50,977	19.14	893	130	USA (321)
Netherlands	1244	73.05	2276	37,658	30.27	607	134	USA (189)
Germany	1188	14.40	2229	30,196	25.41	576	130	USA (226)
Sweden	1002	100.98	1761	25,502	25.47	434	127	USA (147)
South Africa	910	22.64	1597	20,626	22.64	432	125	USA (212)
Israel	860	100.63	1387	18,245	21.22	307	110	USA (198)
The People’s Republic of China	755	0.55	1839	17,299	22.91	355	123	USA (194)
Italy	738	12.17	2117	20,766	28.14	347	132	USA (143)
Spain	695	14.95	1849	15,148	21.80	411	128	USA (94)
Switzerland	655	78.59	1959	32,891	49.99	495	134	USA (200)
New Zealand	608	129.55	1352	32,584	53.59	230	115	USA (88)
Brazil	576	2.77	1578	8440	14.65	211	121	USA (93)
India	571	0.43	1723	11,357	19.89	170	119	USA (101)
Norway	550	105.04	1474	10,126	18.41	225	120	USA (86)
Scotland	548	101.58	1475	15,431	28.11	264	125	England (165)
France	528	7.89	1393	16,876	31.96	265	129	USA (90)
Turkey	512	6.44	1275	5715	11.16	85	112	USA (54)
Finland	456	82.98	1332	12,090	26.51	164	120	USA (59)
Japan	437	3.44	1576	10,483	23.99	127	122	USA (71)
Denmark	367	64.07	1443	11,753	32.02	197	125	USA (71)
Belgium	352	31.04	1338	7884	22.40	208	118	Netherlands (74)
South Korea	331	6.46	1298	4610	13.93	158	116	USA (123)
Wales	330	106.00	1099	8128	24.63	187	106	England (145)
Ireland	280	58.95	1183	9099	32.50	135	107	England (54)
Taiwan (China)	226	9.60	1170	4017	17.77	81	116	USA (54)
Nigeria	220	1.18	1278	3989	18.13	82	120	USA (44)
Iran	215	2.68	1155	2734	12.72	70	113	USA (35)
Northern Ireland	188	100.96	226	2500	13.30	89	41	USA (35)
Portugal	184	17.82	1255	2837	15.42	83	122	England (27)
Austria	175	20.04	1205	5847	33.41	103	105	Germany (43)
Kenya	174	3.59	1285	4498	25.85	149	119	USA (96)
Greece	154	14.30	1243	4155	26.98	68	120	England (32)
Mexico	150	1.18	1271	7784	51.89	86	118	USA (69)
Uganda	150	3.62	1140	6271	41.81	137	114	England (65)
Pakistan	139	0.72	1205	3959	28.48	81	113	USA (40)
Poland	134	3.53	1007	1996	14.90	41	105	England (14)
Malaysia	123	3.94	1128	2260	18.37	60	115	Australia (16)
Saudi Arabia	122	3.78	1161	5737	47.02	57	116	USA (25)
Singapore	111	19.80	1156	6438	58.00	65	116	USA (30)
Thailand	103	1.50	660	1845	17.91	72	96	USA (27)
Egypt	102	1.07	1096	2398	23.51	47	114	Saudi Arabia (18)
Tanzania	95	1.71	1116	3805	40.05	86	120	USA (31)
Ethiopia	94	0.92	1098	3296	35.06	59	114	England (30)
Columbia	92	1.89	1174	3296	35.06	68	122	USA (46)
Croatia	89	21.32	165	751	8.44	24	40	USA (8)
Russia	83	0.58	1174	5585	67.29	53	118	USA (40)
Ghana	78	2.77	1194	5787	74.19	66	114	USA (34)

**Table 5:** Most cited papers.

Rank	Paper	Total Citation	Citations/Year
1	Felitti VJ, Anda RF, Nordenberg D, Williamson DF, Spitz AM, Edwards V, Koss MP, Marks JS. Relationship of childhood abuse and household dysfunction to many of the leading causes of death in adults—The adverse childhood experiences (ACE) study. American Journals of Preventive Medicine. 1998;14:245–258	3356	167.8
2	Lozano R, et al. Global and regional mortality from 235 causes of death for 20 age groups in 1990 and 2010: a systematic analysis for the Global Burden of Disease Study 2010. Lancet. 2012;380:2095–2128	3307	551.2
3	Caspi A, McClay J, Moffitt TE, Mill J, Martin J, Craig IW, Taylor A, Poulton R. Role of genotype in the cycle of violence in maltreated children. Science. 2002;297:851–854	2366	147.9
4	Resnick MD, Bearman PS, Blum RW, Bauman, KE, Harris KM, Jones J, Tabor J, Beuhring T, Sieving RE, Shew M, Ireland M, Bearinger LH, Udry JR. Protecting adolescents from harm—Findings from the National Longitudinal Study on Adolescent Health. JAMA Journal of the American Medical Association. 1997;278:823–832	2335	111.2
5	Nansel TR, Overpeck M, Pilla RS, Ruan WJ, Simons-Morton B, Scheidt P. Bullying behaviors among US youth—Prevalence and association with psychosocial adjustment. JAMA Journal of the American Medical Association. 2001;285:2094–2100	1601	94.2
6	Browne A; Finkelhor D. Impact of child sexual abuse—a review of the research. Psychological Bulletin. 1986;99:66–77	1464	45.8
7	Leventhal T, Brooks-Gunn J. The neighborhoods they live in: The effects of neighborhood residence on child and adolescent outcomes. Psychological Bulletin. 2000;126:309–337	1461	81.2
8	Heim C; Nemeroff CB. The role of childhood trauma in the neurobiology of mood and anxiety disorders: Preclinical and clinical studies. Biological Psychiatry. 2001;49:1023–1039	1408	82.8
9	Bernstein DP, Stein JA, Newcomb MD, Walker E, Pogge D, Ahluvalia T, Stokes J, Handelsman L, Medrano M, Desmond D, Zule W. Development and validation of a brief screening version of the Childhood Trauma Questionnaire. Child Abuse and neglect. 2003;27:69–190	1320	88.0
10	Angold A, Costello EJ, Erkanli A. Comorbidity. Journal of Child Psychology and Psychiatry and Applied Disciplines. 1999;40:57–87	1315	69.2
11	Prince M, Patel V, Saxena S, Maj M, Maselko J, Phillips MR, Rahman A. Global mental health 1—No health without mental health. Lancet. 2007;370:859–877	1213	110.3
12	Durlak JA, Weissberg RP, Dymnicki AB, Taylor RD, Schellinger KB. The Impact of Enhancing Students’ Social and Emotional Learning: A Meta-Analysis of School-Based Universal Interventions. Child Development. 2001;82:405–432	1169	167.0
13	Hayes SC, Wilson KG, Gifford EV, Follette VM, Strosahl K. Experiential avoidance and behavioral disorders: A functional dimensional approach to diagnosis and treatment. Journal of Consulting and Clinical Psychology. 1996;64:1152–1168	1147	52.1
14	Heim C, Newport DJ, Heit S, Graham YP, Wilcox M, Bonsall R, Miller AH, Nemeroff CB. Pituitary-adrenal and autonomic responses to stress in women after sexual and physical abuse in childhood. JAMA Journal of the American Medical Association. 200;284:592–597	1093	60.7
15	Gilbert R, Widom CS, Browne K, Fergusson D, Webb E, Janson S. Child Maltreatment 1 Burden and consequences of child maltreatment in high-income countries. Lancet. 2009;373:68–81	1091	121.2
16	Anda RF, Felitti VJ, Bremner JD, Walker JD, Whitfield C, Perry BD, Dube SR, Giles WH. The enduring effects of abuse and related adverse experiences in childhood—A convergence of evidence from neurobiology and epidemiology. European Archives of Psychiatry and Clinical Neuroscience. 2006;256:174–186	1057	88.1
17	Biederman J, Newcorn J, Sprich S. Comorbidity of attention-deficit hyperactivity disorder with conduct, depressive, anxiety, and other disorders. American Journal of Psychiatry. 1999;148:564–577	1026	38.0
18	Birmaher B, Ryan ND, Williamson DE, Brent DA, Kaufman J, Dahl RE, Perel J, Nelson B. Childhood and adolescent depression: A review of the past 10 years. Journal of the American Academy of Child and Adolescent Psychiatry. 1996;35:1427–1439	997	45.3
19	Dishion TJ, McCord J, Poulin F. When interventions harm—Peer groups and problem behavior. American Psychologist. 1999;54:755–764	956	50.3
20	Brewin CR, Andrews B, Gotlib IH. Psychopathology and early experience—a reappraisal of retrospective reports. Psychological Bulletin. 1993;113:82–98	941	37.6
21	Terr LC. Childhood traumas—an outline and overview. American Journal of Psychiatry. 1991;48:10–20	909	33.7
22	Barber BK. Parental psychological control: Revisiting a neglected construct. Child Development. 1996;67:3296–3319	902	41.0
23	Straus MA, Hamby SL, Finkelhor D, Moore DW, Runyan D. Identification of child maltreatment with the parent-child Conflict Tactics Scales: Development and psychometric data for a national sample of American parents. Child Abuse and Neglect. 1998;22:249–270	860	43.0
24	Evans GW. The environment of childhood poverty. American Journal of Psychiatry. 2004;59:77–92	831	59.4
25	Shonkoff JP, Garner AS. The Lifelong Effects of Early Childhood Adversity and Toxic Stress. Pediatrics. 2012;129:E232-E246	821	136.8
26	Moffitt TE, Caspi A, Harrington H, Milne BJ. Males on the life-course-persistent and adolescence-limited antisocial pathways: Follow-up at age 26 years. Development and Psychopathology. 2002;14:179–207	816	51.0
27	Ceci SJ, Bruck M. Suggestibility of the child witness-a historical review and synthesis. Psychological Bulletin. 1993;113:403–439	801	32.0
28	Dube SR, Anda RF, Felitti VJ, Chapman DP, Williamson DF, Giles WH. Childhood abuse, household dysfunction, and the risk of attempted suicide throughout the life span—Findings from the adverse childhood experiences study. JAMA Journal of the American Medical Association. 2001;286:3089–3096	794	46.7
29	Beithchman JH; Zucker KJ; Hood JE; Dacosta GA; Akman D; Cassavia, E. A review of the long-term effects of child sexual abuse. Child Abuse and Neglect. 1992;16:101–118	791	30.4
30	Bar-on ME, Broughton DD, Buttross S, Corrigan S, Gedissman A, de Rivas MRG, Rich M, Shifrin DL. Children, adolescents, and television. Pediatrics. 2001;107:423–426	772	45.4
31	Shaffer D, Gould MS, Fisher P, Trautman P, Moreau D; Kleinman M; Flory M. Psychiatric diagnosis in child and adolescent suicide. Archives of General Psychiatry. 1996;53:339–348	760	34.5
32	Walker SP, Wachs TD, Gardner JM, Lozoff B, Wasserman GA, Pollitt E, Carter JA. Child development in developing countries 2—Child development: risk factors for adverse outcomes in developing countries. Lancet. 2007;369:145–157	753	68.5
33	Anderson CA, Bushman BJ. Effects of violent video games on aggressive behavior, aggressive cognition, aggressive affect, physiological arousal, and prosocial behavior: A meta-analytic review of the scientific literature. Psychological Science. 2001;12:353–359	749	44.1
34	Finkelhor D; Browne A. The traumatic impact of child sexual abuse—a conceptualization. American Journal of Orthopsychiatry. 1985;55:530–541	740	22.4
35	Salmivalli C, Lagerspetz K, Bjorkqvist K, Osterman K, Kaukiainen A. Bullying as a group process: Participant roles and their relations to social status within the group. Aggressive Behavior. 1996;22:1–15	719	32.7
36	Loftus EF. The reality of repressed memories. American Psychologist. 1993;18:518–537	711	28.4
37	Read, J; van Os, J; Morrison, AP; Ross, CA. Childhood trauma, psychosis and schizophrenia: a literature review with theoretical and clinical implications. Acta Psychiatrica Scandinavica. 2005;112:330–350	706	54.3
38	Herman, JL. Complex PTSD—a syndrome in survivors of prolonged and repeated trauma. Journal of Traumatic Stress. 1992;5:377–391	704	27.1
39	Binder, EB et al. Association of FKBP5 polymorphisms and childhood abuse with risk of posttraumatic stress disorder symptoms in adults. JAMA Journal of the American Medical Association. 2008; 299:1291–1305	690	69.0
40	Olds, DL et al. Long-term effects of home visitation on maternal life course and child abuse and neglect—Fifteen-year follow-up of a randomized trial. JAMA Journal of the American Medical Association. 1997;278:1291–1305	684	32.6
41	Belsky, J. Child maltreatment—an ecological integration. American Psychologist. 1980;35:320–335	670	17.6
42	Molnar, BE; Buka, SL; Kessler, RC. Child sexual abuse and subsequent psychopathology: Results from the National Comorbidity Survey. American Journal of Public Health. 2001;91:753–760	639	37.6
43	McCauley, J et al. Clinical characteristics of women with a history of childhood abuse—Unhealed wounds. JAMA Journal of the American Medical Association 1997;277:1362–1368	628	29.9
44	Kim-Cohen, J. MAOA, maltreatment, and gene-environment interaction predicting children’s mental health: new evidence and a meta-analysis. Molecular Psychiatry. 2006;11:903–913	614	51.2
45	Belsky, J. Etiology of child maltreatment—a developmental ecological analysis. Psychological Bulletin. 1983;114:413–434	611	17.5
46	Putnam, FW. Ten-year research update review: Child sexual abuse. Journal of the American Academy of Child and Adolescent Psychiatry. 2003;42:269–278	609	40.6
47	Margolin, G; Gordis, EB. The effects of family and community violence on children. Annual Review of Psychology. 2000;51:445–479	606	33.7
48	Krug, EG; Mercy, JA; Dahlberg, LL; Zwi, AB. The world report on violence and health. Lancet. 2002;360:1083–1088	601	37.6

## Supplementary Materials

The following are available online at http://www.mdpi.com/1660-4601/15/7/1456/s1, Table S1: Search Strategy, Table S2: Top 100 prolific authors, Table S3: Top 100 prolific countries.
