# Supplementary material for: A Bibliometric Analysis of the Global Research Trend in Child Maltreatment
Source: Int J Environ Res Public Health. 2018 Jul 10;15(7):1456. doi: 10.3390/ijerph15071456 (PMC6069266; doi:10.3390/ijerph15071456)
Supplement: Supplementary file 1 [file ijerph-15-01456-s001.zip › ijerph-324920-SI/Table S1 Search strategy and results.pdf]

Table S1. Search strategy and results.
Article
02052018

	**Terms ("topic")**	**Results**
# 1	TS=(child)	991,587
# 2	TS=(violence)	75,239
# 3	TS=(abuse)	125,579
# 4	TS=(maltreatment)	10,035
# 5	TS=(sexual)	206,564
# 6	TS=(physical)	932,612
# 7	TS=(emotional)	139,431
# 8	TS=(psychological)	190,308
# 9	TS=(neglect)	100,946
# 10	TS=(trafficking)	167,294
# 11	TS=(grooming)	6,441
# 12	#2 AND #1	14,512
# 13	#3 AND #1	28,687
# 14	#4 AND #1	8,140
# 15	#5 AND #3 AND #1	10,357
# 16	#6 AND #3 AND #1	6,576
# 17	#7 AND #3 AND #1	2,969
# 18	#8 AND #3 AND #1	2,911
# 19	#9 AND #1	9,042
# 20	#10 AND #1	4,280
#21	#11 AND #1	235
#22	TS=(animal* NOT human*)	614,835

#23	#21 OR #20 OR #19 OR #18 OR #17 OR #16 OR #15 OR #14 OR #13 OR #12	46,831
#24	(#23 NOT #22) *AND* LANGUAGE: (English) *AND* DOCUMENT TYPES: (Article)	46,458
#25	(#23 NOT #22) *AND* LANGUAGE: (English) *AND* DOCUMENT TYPES: (Article)<br>Refined by: [excluding] DOCUMENT TYPES: (PROCEEDINGS PAPER OR BOOK CHAPTER OR RETRACTED PUBLICATION OR BOOK OR DATA PAPER OR EARLY ACCESS)	43,561
Limits	Type of document: (Article)	
	Language: English	

Review

	**Terms ("topic")**	**Results**
# 26	TS=(child)	**68,022**
# 27	TS=(violence)	**3,832**
# 28	TS=(abuse)	**10,902**
# 29	TS=(maltreatment)	**752**
# 30	TS=(sexual)	**15,407**
# 31	TS=(physical)	**63,255**
# 32	TS=(emotional)	**9,373**
# 33	TS=(psychological)	**17,901**
# 34	TS=(neglect)	**7,018**
# 35	TS=(trafficking)	**12,401**
# 36	TS=(grooming)	**226**
# 37	#27 AND #26	**969**
# 38	#28 AND #26	**2,264**
# 39	#29 AND #26	**592**
# 40	#30 AND #28 AND #26	**866**
# 41	#31 AND #28 AND #26	**541**

# 42	#32 AND #28 AND #26	**257**
# 43	#33 AND #28 AND #26	**337**
# 44	#34 AND #26	**844**
#45	#35 AND #26	**284**
#46	#36 AND #26	**17**
#47	TS=(animal* NOT human*)	**42,498**
#48	#46 OR #45 OR #44 OR #43 OR #42 OR #41 OR #40 OR #39 OR #38 OR #37	**3,680**
#49	(#48 NOT #47) *AND* **LANGUAGE:** (English) *AND* **DOCUMENT TYPES:** (Review)	**3,645**
#50	(#48 NOT #47) *AND* **LANGUAGE:** (English) *AND* **DOCUMENT TYPES:** (Review) **Refined by:** [excluding] **DOCUMENT TYPES:** (BOOK CHAPTER OR EARLY ACCESS OR RETRACTED PUBLICATION)	**3,602**
Limits	Type of document: (Review)	
	Language: English	

Results:

#51	#25 OR #50 **Refined by:** [excluding] **AUTHORS:** (ANONYMOUS)	47,103
