# Supplementary material for: A Bibliometric Analysis of the Global Research Trend in Child Maltreatment
Source: Int J Environ Res Public Health. 2018 Jul 10;15(7):1456. doi: 10.3390/ijerph15071456 (PMC6069266; doi:10.3390/ijerph15071456)
Supplement: Supplementary file 1 [file ijerph-15-01456-s001.zip › ijerph-324920-SI/Table S2 Top 100 the most prolific authors.pdf]

Table S2: Top 100 most prolific authors.

	Authors	records	% of 47103
1	CICCHETTI D	159	0.338
2	FINKELHOR D	116	0.246
3	DUBOWITZ H	96	0.204
4	LAMB ME	96	0.204
5	FERGUSSON DM	87	0.185
6	MILNER JS	83	0.176
7	HORWOOD LJ	77	0.163
8	WIDOM CS	73	0.155
9	GOODMAN GS	71	0.151
10	POWELL MB	70	0.149
11	ROGOSCH FA	68	0.144
12	UTZINGER J	68	0.144
13	BRUNEKREEF B	65	0.138
14	DODGE KA	65	0.138
15	MACMILLAN HL	65	0.138
16	RUNYAN DK	64	0.136
17	TRICKETT PK	61	0.13
18	HEBERT M	60	0.127
19	THOMPSON R	60	0.127
20	BARTH RP	59	0.125
21	LEVENTHAL JM	58	0.123
22	PUTNAM FW	58	0.123
23	TROCME N	56	0.119
24	GRAHAM-BERMANN SA	54	0.115
25	FALLON B	53	0.113
26	JONSON-REID M	53	0.113
27	MCLAUGHLIN KA	52	0.11
28	FISHER PA	51	0.108
29	TOTH SL	51	0.108
30	WRIGHT RJ	50	0.106
31	COHEN JA	48	0.102
32	QUAS JA	48	0.102
33	BIEDERMAN J	47	0.1
34	HEINRICH J	47	0.1
35	JENNY C	47	0.1
36	KLEINMAN PK	47	0.1
37	LITROWNIK AJ	47	0.1
38	VAN IJZENDOORN MH	47	0.1
39	LANDSVERK J	46	0.098
40	MOFFITT TE	46	0.098
41	SVEDIN CG	46	0.098

42	BRENT DA	45	0.096
43	LYON TD	45	0.096
44	KELLEY ML	44	0.093
45	SLEP AMS	44	0.093
46	TONMYR L	44	0.093
47	BLACK MM	43	0.091
48	ELBERT T	43	0.091
49	WALSH K	43	0.091
50	WEKERLE C	43	0.091
51	BAKERMANS-KRANENBURG MJ	42	0.089
52	BENBENISHTY R	42	0.089
53	CHRISTIAN CW	42	0.089
54	WOLFE DA	42	0.089
55	CHAFFIN M	41	0.087
56	KIRISCI L	41	0.087
57	DRAKE B	40	0.085
58	ENGLISH DJ	40	0.085
59	KIM J	40	0.085
60	NAJMAN JM	40	0.085
61	BERGER RP	39	0.083
62	CASPI A	39	0.083
63	FELDMAN KW	39	0.083
64	SAUNDERS BE	38	0.081
65	TAYLOR J	38	0.081
66	TREMBLAY RE	38	0.081
67	CUMMINGS EM	37	0.079
68	HERRENKOHL TI	37	0.079
69	HERSHKOWITZ I	37	0.079
70	KEMP AM	37	0.079
71	LUTZKER JR	37	0.079
72	PUNAMAKI RL	37	0.079
73	RIVARA FP	37	0.079
74	TARTER RE	37	0.079
75	WILENS TE	37	0.079
76	FARRINGTON DP	36	0.076
77	HORWITZ SM	36	0.076
78	HOWELL KH	36	0.076
79	JOURILES EN	36	0.076
80	MCDONALD R	36	0.076
81	RYAN JP	36	0.076
82	SEEDAT S	36	0.076
83	TURNER HA	36	0.076
84	AFIFI TO	35	0.074
85	BIRMAHER B	35	0.074

86	FARAONE SV	35	0.074
87	HJERN A	35	0.074
88	HOEK G	35	0.074
89	KASSAM-ADAMS N	35	0.074
90	MANNARINO AP	35	0.074
91	SUNYER J	35	0.074
92	ZEANAH CH	35	0.074
93	BETANCOURT TS	34	0.072
94	CROUCH JL	34	0.072
95	DEBLINGER E	34	0.072
96	STEIN DJ	34	0.072
97	BOXER P	33	0.07
98	BRADLEY B	33	0.07
99	FONAGY P	33	0.07
100	FREISTHLER B	33	0.07
