# Supplementary material for: A Bibliometric Analysis of the Global Research Trend in Child Maltreatment
Source: Int J Environ Res Public Health. 2018 Jul 10;15(7):1456. doi: 10.3390/ijerph15071456 (PMC6069266; doi:10.3390/ijerph15071456)
Supplement: Supplementary file 1 [file ijerph-15-01456-s001.zip › ijerph-324920-SI/Table S3 Top 100 the most productive countries.pdf]

Table S3: Top 100 the most productive countries.

	Countries/Regions	records	% of 47090
1	USA	26377	56.0
2	ENGLAND	4675	9.9
3	CANADA	3282	7.0
4	AUSTRALIA	2664	5.7
5	NETHERLANDS	1244	2.6
6	GERMANY	1188	2.5
7	SWEDEN	1002	2.1
8	SOUTH AFRICA	910	1.9
9	ISRAEL	860	1.8
10	PEOPLES R CHINA	755	1.6
11	ITALY	738	1.6
12	SPAIN	695	1.5
13	SWITZERLAND	655	1.4
14	NEW ZEALAND	608	1.3
15	BRAZIL	576	1.2
16	INDIA	571	1.2
17	NORWAY	550	1.2
18	SCOTLAND	548	1.2
19	FRANCE	528	1.1
20	TURKEY	512	1.1
21	FINLAND	456	1.0
22	JAPAN	437	0.9
23	DENMARK	367	0.8
24	BELGIUM	352	0.7
25	SOUTH KOREA	331	0.7
26	WALES	330	0.7
27	IRELAND	280	0.6
28	TAIWAN	226	0.5
29	NIGERIA	220	0.5
30	IRAN	215	0.5
31	NORTH IRELAND	188	0.4
32	PORTUGAL	184	0.4
33	AUSTRIA	175	0.4
34	KENYA	174	0.4
35	GREECE	154	0.3
36	MEXICO	150	0.3
37	UGANDA	150	0.3
38	PAKISTAN	139	0.3
39	POLAND	134	0.3
40	MALAYSIA	123	0.3
41	SAUDI ARABIA	122	0.3

42	SINGAPORE	111	0.2
43	THAILAND	103	0.2
44	EGYPT	102	0.2
45	TANZANIA	95	0.2
46	ETHIOPIA	94	0.2
47	COLOMBIA	92	0.2
48	CROATIA	89	0.2
49	RUSSIA	83	0.2
50	GHANA	78	0.2
51	ROMANIA	72	0.2
52	BANGLADESH	71	0.2
53	CHILE	66	0.1
54	CZECH REPUBLIC	63	0.1
55	SERBIA	59	0.1
56	JORDAN	58	0.1
57	MALAWI	52	0.1
58	VIETNAM	52	0.1
59	HUNGARY	49	0.1
60	SLOVENIA	48	0.1
61	INDONESIA	46	0.1
62	PERU	46	0.1
63	PHILIPPINES	46	0.1
64	SRI LANKA	45	0.1
65	ZIMBABWE	43	0.1
66	LEBANON	42	0.1
67	JAMAICA	41	0.1
68	ICELAND	39	0.1
69	ARGENTINA	37	0.1
70	NEPAL	36	0.1
71	COTE IVOIRE	35	0.1
72	ZAMBIA	35	0.1
73	HONG KONG	34	0.1
74	U ARAB EMIRATES	34	0.1
75	CAMEROON	33	0.1
76	ESTONIA	33	0.1
77	SLOVAKIA	32	0.1
78	SENEGAL	31	0.1
79	CYPRUS	30	0.1
80	BOTSWANA	29	0.1
81	RWANDA	28	0.1
82	CAMBODIA	26	0.1
83	LITHUANIA	26	0.1
84	KUWAIT	24	0.1
85	QATAR	24	0.1

86	IRAQ	23	0.0
87	MOZAMBIQUE	23	0.0
88	PAPUA N GUINEA	20	0.0
89	BOSNIA HERCEG	19	0.0
90	ECUADOR	19	0.0
91	LUXEMBOURG	19	0.0
92	TRINID TOBAGO	19	0.0
93	URUGUAY	18	0.0
94	BULGARIA	16	0.0
95	BURUNDI	16	0.0
96	MALI	16	0.0
97	SUDAN	16	0.0
98	YEMEN	16	0.0
99	BURKINA FASO	15	0.0
100	OMAN	15	0.0
